# Cognitive and neural signatures of the APOE E4 allele in mid-aged adults

**DOI:** 10.1016/j.neurobiolaging.2014.01.145

**Published:** 2014-07

**Authors:** Simon Evans, Nicholas G. Dowell, Naji Tabet, Paul S. Tofts, Sarah L. King, Jennifer M. Rusted

**Affiliations:** a School of Psychology, University of Sussex, Brighton, UK; b Brighton and Sussex Medical School (BSMS), Clinical Imaging Sciences Centre, Brighton, UK; c Brighton and Sussex Medical School (BSMS), Institute of Postgraduate Medicine, Brighton, UK

**Keywords:** Alzheimer's disease, Imaging, Memory, Attention, APOE, Aging

## Abstract

The apolipoprotein E (APOE) e4 allele is strongly associated with increased risk of cognitive impairments in older adulthood. There is also a possible link to enhanced cognitive performance in younger adults, and the APOE e4 allele may constitute an example of antagonistic pleiotropy. The aim of this work was to investigate the cognitive and neural (functional) effects of the APOE e4 allele during mid-age (45–55 years), where a transition toward cognitive deficit might be expected. APOE e4 carriers (e4+) were compared with non-e4 carriers (e4−) on tasks of sustained and covert attention and prospective memory, and functional magnetic resonance imaging data acquired. Performance by e4+ was equivalent or better than e4− on all 3 tasks, although performance benefits were less pronounced than in youth. Neurally, e4+ showed less task-related recruitment of extrastriate and parietal areas. This became more evident when neural activation data were compared with that of young adults acquired in a parallel study. As expected, mid-age participants showed more diffuse neural activation. Notable was the fact that e4+ showed a relative inability to recruit parietal regions as they aged. This was coupled with a tendency to show greater recruitment of frontal regions, and underactivation of extrastriate visual regions. Thus, mid-age e4+ show a pattern of neural recruitment usually seen later in life, possibly reflecting the source of an accelerated aging profile that describes the e4 genotype.

## 1 Introduction

Apolipoprotein E (APOE) is a polymorphic protein involved in neurogenesis, plasticity, and repair, which has 3 allelic variants (e2, e3, and e4) (Mahley, 1988). The e4 allele is the best-established genetic risk factor for Alzheimer's disease (AD), most likely because of its effects on amyloid beta metabolism (Kim et al., 2009). The e4 allele is also associated with poorer recovery from brain trauma (Zhou et al., 2008) and an increased incidence of mild cognitive impairment (Lopez et al., 2003). In normally aging older adults, the e4 allele adversely affects cognitive performance. Older e4 carriers (e4+) are outperformed by non-e4 carriers (e4−), particularly on tests of memory function (Reinvang et al., 2009; Zehnder et al., 2009). APOE status influences the rate of decline in cognitive performance across the life span (Deary et al., 2002; Schiepers et al., 2012). Differences in brain morphology have been associated with these effects; e4+ show disrupted white matter integrity (Heise et al., 2011), and decreased hippocampal and amygdala volume in some studies (Honea et al., 2009).

In spite of the negative consequences associated with the e4 allele in later life, there is some evidence that paradoxically the e4 allele may confer certain cognitive advantages in youth (Marchant et al., 2010; Rusted et al., 2013), and therefore, represent an example of antagonistic pleiotropy (Han and Bondi, 2008), where a gene impacts fitness differently across the life span. This hypothesis would predict that during mid-age, e4+ will transition from being cognitively advantaged to being cognitively disadvantaged. This age point might represent the best opportunity for intervention strategies to protect e4+ against imminent cognitive decline, but relatively little is known about APOE-related cognitive and functional activation differences at mid-age. A study by Flory et al. (2000) found impaired learning and memory performance in mid-aged e4+, although their definition of mid-age encompassed an age span of 24–60 years, which is probably too broad to provide meaningful information regarding the transition phase in e4+. Similarly, a longitudinal study of midlife cognition, with an extended age range of 33–85 years, reported that attentional scaling was compromised in e4+ (Greenwood et al., 2005b). Given that cognitive deficits in e4+ have been detected as early as 60 years (Greenwood et al., 2000), studies examining APOE effects on cognitive performance at mid-age should focus on a narrower age range that might better isolate the transition into poorer age-related cognitive change in e4+.

Imaging techniques have also been used to examine neural differences in e4+ at mid-age. Using positron-emission tomography, cognitively normal e4+ aged 50 to 65 years have been shown to have abnormally low cerebral metabolic rates for glucose in brain regions where AD patients also show reductions in metabolism; these controls also show widespread reductions in metabolic rate across prefrontal regions, above and beyond that seen in AD (Reiman et al., 1996). This could reflect accelerated aging processes, and in support of this notion, mid-aged e4+ have faster declines in metabolism in these regions when measured longitudinally (Reiman et al., 2001). Similar reductions are present in younger (aged 20–39 years) e4+ (Reiman et al., 2004), however, making it difficult to map these metabolic changes onto the cognitive findings. Functional magnetic resonance imaging (fMRI) studies support the notion of abnormal aging in brain responses in e4+. Using an encoding memory task, Filippini et al. (2011) found that whereas e4− showed greater activity in the temporal and cerebellar regions in mid-aged and older adults (50–78 years) compared with younger (20–35 years) participants, e4+ instead showed decreased activity. This suggests that e4+ fail to recruit normal age-related compensatory processes as they move through mid-age (though once again, the extended age range precludes any firm conclusions specific to mid-age individuals). Because young adult e4+ tend to show greater activations during memory tasks (Dennis et al., 2010; Filippini et al., 2009), it is possible that this increased activity in youth might be responsible for neurophysiological changes that accelerate declines in brain function.

In earlier work, we explored neural activation patterns and performance differences in young adult e4+ and e4−. In the 18–28 years age range, we showed that e4+ can manifest an advantage on specific cognitive measures. Specifically, we reported that e4+ generated more words in the early stages of a verbal fluency task, and were faster at the ongoing component of a prospective memory (PM) task (Marchant et al., 2010). e4+ outperformed their e4− peers on a sustained attention (rapid visual information processing [RVIP]) task, and in an attentional switching task they showed less of a switching “cost” when an incongruent cue is presented (Rusted et al., 2013). In terms of functional activity, young adult e4+ demonstrated greater parietal recruitment during attentional tasks, suggesting that they exhibit broader attentional capture mediated by enhanced parietal involvement (Rusted et al., 2013). Even where there was equivalent performance between young adult e4+ and e4−, on PM tasks, neural activation patterns were significantly different between groups (Evans et al., 2013b). This could reflect differences in resting state activation in the default mode network, which have been reported by some studies (Filippini et al., 2009; Westlye et al., 2011).

In the present work our principle aim was to extend these investigations into a mid-age cohort of e4+ and e4−, using a narrow age range of 45–55 years. We used a battery of cognitive tasks that included measures of attention and PM, for some of which fMRI data were acquired. Because many studies of APOE and cognition have used tasks that specifically target the medial temporal lobe (MTL) function, we chose tasks that instead recruit frontal and parietal regions, to investigate effects of APOE in regions beyond the MTL. Using our published data from young adults (Evans et al., 2013b; Rusted et al., 2013) we carried out analyses to investigate age by genotype interactions in cognitive performance and neural activation patterns. For both the cognitive and neural data, we first analyzed all data (from both young and mid-aged participants) using analysis of variance (ANOVA). This was followed by analyses comparing mid age groups only, so as to characterize genotype effects at this age point (similar analyses in the young age group are published elsewhere (Evans et al., 2013b; Rusted et al., 2013). Finally, in the case of the neural data, we contrasted between age groups separately for each genotype. A masking strategy was used to localize age related changes in activity that was genotype-specific. This strategy specifically addressed the hypothesis that e4+ would show neural activity patterns consistent with an accelerated aging profile. We firstly predicted that volunteers would show greater recruitment of task-relevant brain regions at mid-age than in young adulthood, as age-related changes require greater activation to sustain performance. Secondly, we predicted that at mid age e4+ would show recruitment of regions outside the typical network engaged by the task, as processes of dedifferentiation and frontal compensation (usually seen in older adults) are engaged by e4+.

## 2 Methods

### 2.1 Participants

#### 2.1.1 Participants and genotyping procedures

Healthy young participants (98) were recruited (aged 20 ± 2 years, range 18–30 years, 64 women, 34 men), and healthy middle-aged participants (78) were recruited (range 43–58 years, 44 women, 34 men), all right-handed. Volunteers were excluded from the study for untreated high blood pressure, cardiac pathology, a history of psychiatric or neurologic illness, current use of psychoactive medication, and presence of metallic implants including bridges and braces, or tattoos above the shoulder. All participants were non-smokers (and had been for at least 5 years), had a body mass index within the normal range and had English as their first language.

Initial screening involved signing of written informed consent (following procedures approved by the University of Sussex, Schools of Psychology and Life Sciences Research Ethics Committee), and cheek swab samples were collected for DNA analysis from each participant. APOE genotypes were determined by KBiosciences (Hoddesdon, UK; www.kbioscience.co.uk), using their own system of fluorescence-based competitive allele-specific polymerase chain reaction (KASPar). Two APOE single-nucleotide polymorphisms (SNPs) rs429358 and rs7412 allowed identification of the three major APOE alleles (e2, e3, and e4).

#### 2.1.2 Young age group

From the samples, 9 participants were heterozygous e2 and excluded from the study. Fifty participants were identified as e4−, of which 20 were selected randomly as our control group representing the genotype most frequent in the general population (Rebeck et al., 1993). The remaining 34 volunteers were e4+, from which we randomly selected 21 to be participants to enter our experimental group. This group included 2 participants that were homozygous e4 carriers.

#### 2.1.3 Mid-age group

From the anonymized samples, 9 participants were found to be heterozygous e2. 47 participants were identified as e4−, and the remaining 22 volunteers were e4+. All participants who coded as e4+ and a matched number of randomly selected participants who coded for e4− were invited to take part in the remainder of the study. 22 individuals from the e4− group, and 17 individuals from the e4+ group returned; this group included 3 participants that were homozygous e4 carriers.

Both volunteers and researchers were blinded to participant genotype; further, the image acquisition and analysis were conducted under double-blind procedures (i.e., all preprocessing was completed before group assignment was revealed). Demographics are shown in Table 1.

### 2.2 Materials

#### 2.2.1 IQ

The National Adult Reading Test, NART (Nelson et al., 1991) estimates verbal IQ. Participants read aloud 50 irregular words and errors in pronunciation are recorded.

#### 2.2.2 Episodic memory

Immediate free recall provides an episodic memory score. Participants are presented with 20 unrelated words at a rate of 1 word every 2 seconds in a computerized sequence; they are then prompted for immediate written recall.

#### 2.2.3 RVIP

The 6-minute RVIP task (based on Wesnes and Warburton (1983)) is a computerized task. Volunteers monitored a continuous stream of digits, presented at a rate of 80 digits per minute, and pressed a response button when 3 odd or 3 even digits appeared in succession. Eight such target strings occurred in each 1-minute time bin. Correct detections of targets were recorded within a 1500 ms window following the onset of the third digit in the target sequence. Number of correct detections, average latency to correct detections and the number of false alarms (responses to nontargets) were recorded.

#### 2.2.4 PM task

The ongoing task involved simply sorting computerized images of 52 regular playing cards displayed in random sequential order on-screen. Card faces were displayed for 1 second, after which the card back was displayed for 2 seconds plus a variable jitter between 0.1 and 1 second (mean: 0.5 seconds, standard deviation: 0.24 seconds). Using a 4 button-box in their right hand, volunteers were instructed to press button 1 for HEART cards, button 2 for SPADES (“sort” trials), and make no response to CLUBS or DIAMONDS (“withhold” trials). Volunteers were instructed to respond as quickly as possible. At each of the scanning sessions, volunteers practiced the ongoing sort task before entering the scanner, and were then additionally given the prospective memory instruction: they were asked to respond by pressing button 3 for any occurrence of a number “7” card independent of suit (the PM intention). Thus, when in the scanner, participants performed the ongoing task as practiced, but with the additional PM instruction. Presentation of any “7” card constituted a PM trial. Within each scanner session, each volunteer sorted 8 decks of cards with the PM intention over a total period of approximately 15 minutes. Thus, there were a total of 416 trials, of which 32 were PM trials, 192 were “sort” trials and 192 were “withhold” trials. Accuracy and reaction times (RT) to sort and PM trials were recorded.

#### 2.2.5 Covert attention (CA) task

The CA task (based on Thiel et al., 2004) is a computerized task. In each trial, participants fixate a small diamond in the center of the screen; after 3000 ms 1 side of the diamond changes color, cueing that side of the screen. The target is then displayed to either the left or right of the diamond. The cue is predictive in 70% of trials (cue congruent), and incorrect for the remaining 30% of trials (cue incongruent). Participants indicate with a left or right button response which side of the screen the target appears. Half of each of the 184 congruent and 80 incongruent trials occur with a short cue-target interval (500 ms) and half with a longer cue-target interval (700 ms). In addition to the cued trials, 36 catch trials (where the cue was not followed by a target) and 74 null trials (where no cue or target occurred) are included across the session to prevent response habituation. Thus, there were 374 trials in total. Trial types occurred randomly with the limitation that only congruent or null trials were shown in the first 5 trials and there were no consecutive null trials.

### 2.3 Procedure

Recalled volunteers attended 3 sessions. The first session comprised baseline measures of IQ (NART) and episodic memory (immediate verbal free recall). These were followed by the RVIP task. Finally, participants were familiarized with the CA task. In 2 subsequent scanning sessions (approximately 1 week apart), volunteers participated in a double–blind pharmacologic manipulation, in 1 session self-administering placebo and in the other, nicotine (as nasal sprays), 18–20 minutes before imaging. The sessions were identical and their order randomized and counterbalanced.

The pharmacologic manipulation was designed to explore the effects of nicotine administration on performance, and is reported in a separate paper (Evans et al., 2013a). In this report, analysis was completed using data from the placebo sessions only. After the final session all participants were verbally debriefed and compensated.

### 2.4 Design

Task analyses were completed using data from the placebo sessions only; nevertheless, ANOVA included order of sessions (i.e., whether the placebo session was the first or second scanning session) as a factor.

#### 2.4.1 RVIP task

Number of correct detections, average latency to correct detections and the number of false alarms (responses to nontargets) were analyzed separately using ANOVA. Time bin (6 levels: minutes 1–6) was the within-subject factor; genotype (2 levels: e4+, e4−) the between subject factors.

#### 2.4.2 PM task

Accuracy and RT data were analyzed for PM and for ongoing trials. ANOVA examined effects of genotype (2 levels: e4+, e4−,) and session order (2 levels: first or second scanner session) in a mixed design.

#### 2.4.3 CA task

The “validity effect” is the RT cost associated with the presence of an incongruent cue; it is measured by the difference between mean RTs to the incongruent and congruent conditions. Preliminary ANOVA examined effects of cue-target interval (2 levels: short, long); there were no effects or interactions related to cue-target interval, and so data were collapsed across this factor, for simplicity. Thus, ANOVA involved genotype (2 levels: e4+, e4−) and session order (2 levels: first or second scanner session) in a mixed design.

### 2.5 fMRI recording and analysis

fMRI datasets sensitive to BOLD contrast were acquired at 1.5 T. Standard preprocessing steps were applied; fMRI data were analyzed with the standard hierarchal model approach used in SPM8. Further information can be found in the Supplementary Data. Gender was added as a covariate to control for possible effects.

Analyses are presented at a threshold of *p* < 0.05 FWE-corrected. Small volume corrections, (where appropriate, based on the APOE literature) are also reported at *p* < 0.05 FWE-corrected. A priori regions of interest included areas where differences between e4+ and e4− have been reliably demonstrated (hippocampal formation, superior parietal regions). Regions of interest were defined using the Wake Forest University PickAtlas. Anatomic localization of clusters was performed using the Talairach Daemon (University of Texas, USA), and the anatomy toolbox for SPM (Eickhoff et al., 2005). Exclusive masks, where used, were thresholded at *p* < 0.05 uncorrected.

## 3 Results

### 3.1 Comparison of cognitive performance between age groups

#### 3.1.1 RVIP task

##### 3.1.1.1 Exclusions

Two young age volunteers and 1 mid-age volunteer were excluded because their accuracy for correct detections was comparable to their false alarm rate. For the remaining volunteers, correct detection rate, latencies, and false alarms are presented in Table 2.

##### 3.1.1.2 Correct detections of target sets

There was no effect of age, and no age by genotype interaction. There was a trend toward a main effect of genotype (F [1, 77] = 3.03, *p* = 0.086, 2-tailed), with e4+ scoring more hits. There was also a main effect of time (F [1, 77] = 3.03, *p* = 0.086) and a time by genotype interaction (F [1, 77] = 2.38, *p* = 0.038).

##### 3.1.1.3 RTs to correct detections

There were no effects of age or genotype. There was a trend toward an age by genotype interaction (F [1, 77] = 2.84, *p* = 0.096, 2-tailed), with young e4+ responding faster than e4−, and mid-aged e4+ responding slower than e4−.

##### 3.1.1.4 False alarm rate

There was no main effect of age. There was a trend toward a main effect of genotype (F [1, 77] = 2.99, *p* = 0.088) with e4+ making fewer false alarms. There was no age by genotype interaction.

#### 3.1.2 PM Task

##### 3.1.2.1 Ongoing trials

On accuracy there were no effects of age or genotype. On RTs, there was a main effect of age (F [1, 75] = 10.93, *p* = 0.002) with mid-age participants being slower. There was also a main effect of genotype (F [1, 75] = 5.26, *p* = 0.025), with e4+ being slower than e4−. There were no interactions or effects of session order.

##### 3.1.2.2 PM trials

On accuracy, there were no effects of age or genotype. There was an age by genotype interaction (F [1, 75] = 6.20, *p* = 0.015), with mid-age e4+ detecting more, and mid-age e4− detecting less, relative to the young adult group. There was also a main effect of session order (F [1, 75] = 30.11, *p* < 0.001), with accuracy higher in session 2 (mean accuracy for session 1 = 24.3 [4.07], session 2 = 28.5 [3.13]), but no interactions. On RTs, there was a main effect of age (F [1, 75] = 4.83, *p* = 0.031) with mid-age participants being slower. There was also a main effect of genotype (F [1, 35] = 7.23, *p* = 0.009), with e4+ being slower. There were no interactions.

#### 3.1.3 CA Task

##### 3.1.3.1 Exclusions

One young age volunteer (e4+) and 3 mid-age volunteers were excluded from analysis (all e4−) for not performing the task correctly. After these exclusions, remaining volunteers exceeded 90% correct performance (Table 2).

On this task there was a main effect of age (F [1, 64] = 12.84, *p* = 0.001), with mid-age participants showing a larger validity effect. There were no main effects of genotype or session order, and no interactions. Examining the raw data pointed to a possible outlier in the mid-age group, whose reaction times to incongruent trials were more than 3 standard deviations higher than the mean for the mid-age group. Excluding this participant, the ANOVA demonstrated a significant main effect of genotype (F [1, 70] = 4.44, *p* = 0.039), with e4+ showing a smaller validity effect.

### 3.2 Cognitive performance in mid-age group

#### 3.2.1 RVIP task

##### 3.2.1.1 Correct detections of target sets

**Fig. 1:**
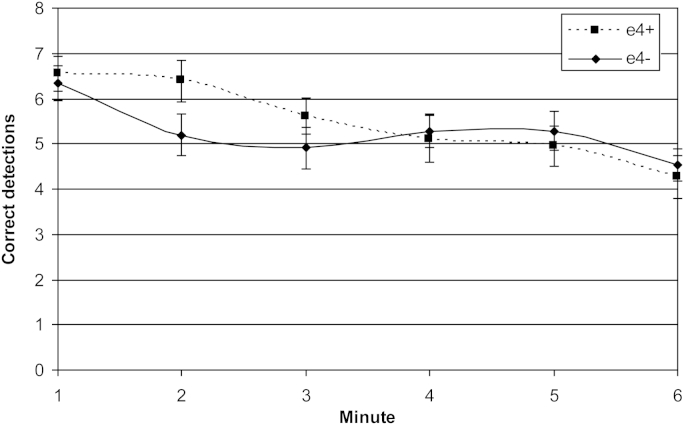
Mid-age group. RVIP task. Mean number of correct detections by genotype across the six 1-minute time bins. Error bars show standard errors on the means. Abbreviation: RVIP, rapid visual information processing.

Fig 1 shows a main effect of time on correct detections (F [1, 37] = 43.6, *p* < 0.001), reflecting a decreasing hit rate over the 6 minutes of the task. There was a time by genotype interaction (F [1, 37] = 4.43, *p* = 0.042). During minute 2, e4+ scored significantly more hits than e4− (F [1, 37] = 3.36, *p* = 0.038, 1-tailed test).

##### 3.2.1.2 RTs to correct detections

There was no main effect of time on latency (F [1, 36]= 0.02, *p* = 0.89), no time by genotype interaction (F [1, 36]= 0.29, *p* = 0.59), and no significant difference in reaction time between e4+/e4− (F [1, 37] = 2.40, *p* = 0.13).

##### 3.2.1.3 False alarm rate

There were no effects of time or genotype and no time by genotype interactions.

#### 3.2.2 PM task

Four volunteers (2 e4+, 2 e4−) completed the RVIP but did not proceed to the imaging phase of the study. Summary data are shown in Table 3.

##### 3.2.2.1 Ongoing trials

There were no genotype effects on accuracy. On reaction times, e4+ were significantly slower than e4− (F [1, 35] = 5.78, *p* = 0.018). There were no effects, or interactions with, session order.

##### 3.2.2.2 PM trials

e4+ were significantly more accurate than e4− (F[1, 35] = 6.33, *p* = 0.017). There was a significant effect of session order (F [1, 35] = 8.94, *p* = 0.005) with accuracy higher in session 2 (mean accuracy for session 1 = 24.7 [4.65], session 2 = 28.1 [3.61]) but no interactions. On reaction times, e4+ were significantly slower than e4− (F [1, 35] = 5.02, *p* = 0.032). There were no effects of, or interactions with, session order.

#### 3.2.3 CA task

The validity effect was analyzed by genotype and session order. ANOVA revealed no main effect of genotype (F [1, 29] = 0.343, *p* = 0.562). There was no effect, or interactions with, session order.

### 3.3 fMRI analysis

#### 3.3.1 PM task

##### 3.3.1.1 Comparison between age groups

**Fig. 2:**
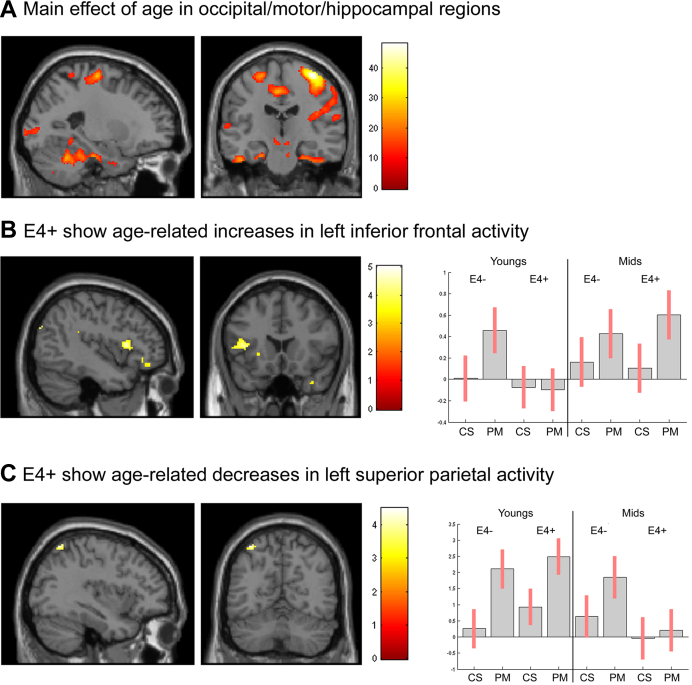
PM task. (A) Main effect of age on all trial types for all participants (B) On PM trials, mid-age e4+ showed greater activity in left inferior frontal compared with young e4+, whereas e4− do not. Associated parameter estimates and 90% confidence intervals are shown for PM and Card Sort (CS) trials. (C) On PM trials, mid age e4+ showed decreased activity in left superior parietal compared with young e4+, whereas e4− showed no difference, as illustrated by the associated parameter estimates. Abbreviation: PM, prospective memory.

Across all trial types, a main effect of age was seen in right Area 6 (x = 42, y = −12, z = 58, *p* < 0.001 FWE-corrected, k = 401) and right IPC (x = 38, y = −82, z = 34, *p* = 0.001 FWE-corrected, k = 49), see Fig 2A. For PM trials only, a main effect of age was seen in right Area 6 (x = 36, y = −20, z = 64, *p* < 0.001 FWE-corrected, k = 536), left parahippocampus (subiculum) (x = −22, y = −20, z = −24, *p* = 0.001 FWE-corrected, k = 134), and right IPC (x = 38, y = −82, z = 34, *p* < 0.001 FWE-corrected, k = 106). In all cases, effects were driven by greater activity in mid-age participants. There were no main effects of genotype, and no age by genotype interactions.

##### 3.3.1.2 Genotype differences at mid age

Across all trial types (sort, withhold, or PM) there were no main effects of genotype, and no interactions between genotype and trial type. On PM trials only, a main effect of genotype was present in extrastriate cortex (x = 16, y = −86, z = 10, *p* = 0.024 FWE-corrected, k = 3) and in right SPL (x = 32, y = −70, z = 56, *p* = 0.038 FWE-corrected following SVC for bilateral SPL, k = 41). An active cluster was also present in left SPL (x = −34, y = −64, z = 58, k = 22) although not significant at *p* < 0.05 FWE-corrected. In both cases, effects were because of reduced activity in e4+.

##### 3.3.1.3 Genotype-specific effects between age groups

To examine genotype-specific effects of aging, the contrasts (mid PM > youngs PM) and (mid PM < youngs PM) were constructed separately for e4− and e4+, and then used as exclusive masks to isolate activity that was genotype-specific.

On PM trials, the contrast (e4+ mid PM > e4+ youngs PM) masked with (e4− mid PM > e4− Youngs PM) revealed activity specific to e4+ in left inferior frontal gyrus (x = −44, y = 16, z = 10, *p* = 0.034 FWE-corrected, k = 172) and left parahippocampus (CA) (x = −28, y = −26, z = 20, *p* = 0.004 FWE-corrected after SVC for bilateral parahippocampi, k = 63). Activity was also present in left orbitofrontal cortex (x = −36, y = 33, z = −10, *p* < 0.001 uncorrected, k = 65), Fig 2B.

The opposite contrast (e4+ mid PM < e4+ youngs PM) masked with (e4− mid PM < e4− youngs PM) revealed activity specific to e4+ in left superior parietal (x = −36, y = −62, z = 60, *p* = 0.018 FWE-corrected after SVC for bilateral SPL, k = 30), Fig 2C.

The reverse masking strategy where results for e4− were masked with e4+ to determine any age-related differences in activity occurring in e4−, but not e4+, yielded no results.

##### 3.3.1.4 Correlations between BOLD activity and cognitive performance

To investigate the significance of the genotype-specific neural activations described previously, activation intensities specific to PM trials were extracted for each mid-age participant in left inferior frontal gyrus, left parahippocampus, and left superior parietal using the coordinates of peak activation identified previously. In left inferior frontal gyrus, activation correlated with PM accuracy for e4+ (r = 0.619, *p* = 0.008) but not for e4− (r = 0.276, *p* = 0.252). There was no significant difference between the r-values of these 2 correlations, using a Fisher r-to-z transformation (z = 1.2, *p* = 0.23). There was no correlation in either genotype with PM RT. In parahippocampus and left superior parietal, there were no correlations with PM accuracy or RT.

##### 3.3.1.5 Parametric modulation by reaction time (PM trials)

In a separate analysis, the reaction time for each Sort and PM trial was included as a parametric modulator, and modeled as a separate column in the design matrix. A significant main effect of modulation by PM reaction time was observed in left Area 6 (x = −20, y = −12, z = +56, k = 1, *p* = 0.028 FWE-corrected). Applying the contrast e4− > e4+ to the modulation revealed activity in extrastriate cortex (x = −6, y = −78, z = +22, k = 51, *p* = 0.026 FWE–corrected after S.V.C for cuneus). Applying the reverse contrast (e4+ < e4−) did not show any effects.

#### 3.3.2 CA task

##### 3.3.2.1 Comparison between age groups

**Fig. 3:**
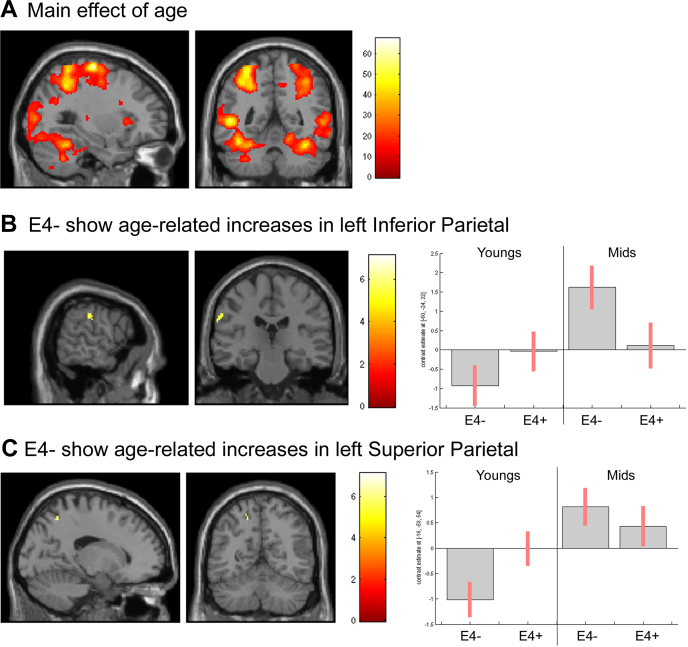
CA task. (A) Main effect of age on all trial types. (B) Examining activity related to the validity effect showed genotype-specific effects between young- and mid-age. E4− showed age-related recruitment of left IPC and (C) left SPL, whereas E4+ did not. Bar plots show associated parameter estimates and 90% confidence intervals for each region. Abbreviation: CA, covert attention.

Neural activity relating to the validity effect was analyzed by contrasting incongruent and congruent conditions. A main effect of age was seen in left Area 6, extending into left inferior and superior parietal lobules (x = −35, y = −32, z = 60, *p* < 0.001 FWE-corrected, k = 1490), right cerebellum (x = 27, y = −65, z = −12 *p* < 0.001 FWE-corrected, k = 655), left cerebellum (x = −41, y = −63, z = −11, *p* < 0.001 FWE-corrected, k = 611), right inferior frontal (x = 44, y = 12, z = 35, *p* = 0.001 FWE-corrected, k = 108), and left SMA (x = −3, y = −8, z = 62, *p* < 0.001 FWE-corrected, k = 75), Fig 3A. In all cases, effects were driven by greater activity in mid-age participants. There were no main effects of genotype, and no age by genotype interactions.

##### 3.3.2.2 Genotype differences at mid age

For the validity effect, no main effects of genotype were observed.

##### 3.3.2.3 Genotype-specific effects of age

For the validity effect, the contrasts (mid > youngs) and (mid < youngs) were constructed separately for e4− and e4+. When results for e4+ were then masked with that of e4− to determine where age-related differences in activity occur in e4+, but not e4−, no significant findings emerged.

Results for e4− were then masked with that of e4+ to determine where age-related differences in activity occur in e4−, but not e4+.

The contrast (e4− mid > e4− young) masked with (e4+ mid > e4+ youngs) revealed activity specific to e4− in left area 6 (x = −55, y = 6, z = 33, *p* < 0.001 FWE-corrected, k = 90), left V5/V5 (x = −41, y = −89, z = 13, *p* = 0.002 FWE-corrected, k = 83), right cerebellum (x = 8, y = −79, z = −34, *p* = 0.002 FWE-corrected, k = 23), left IPC (x = −61, y = −30, z = 35, *p* = 0.019 FWE-corrected, k = 22), and left SPL (x = −17, y = −62, z = 60, *p* = 0.004 FWE-corrected, k = 7), Fig. 3B and C.

The opposite contrast (e4− mid < e4− youngs) masked with (e4+ mid < e4+ youngs) revealed no activity.

## 4 Discussion

In this study, we investigated cognitive performance in mid-aged e4+ and e4− volunteers, and genotype differences in neural activation patterns between youth and mid age individuals. We used tests of sustained and covert attention and prospective memory. Contrary to cognitive performance impairments reported in some earlier studies that had used a much wider age range than we used (Flory et al., 2000; Greenwood et al., 2005a), we found that our 45–55 year old mid-aged e4+ performed on these tasks as well as, or better than, their e4− peers.

On the sustained attention (RVIP) task, mid-aged e4+ outperformed e4− at the trend level. These e4+ advantages were not as pronounced as those we have previously reported in the young adult group, where e4+ outperformed e4− across each minute of the task (Rusted et al., 2013). Interestingly, in the combined analysis which included previously acquired data from a sample of young adults, a trend toward an age by genotype interaction was observed on reaction times, with e4+ showing a greater relative slowing of reaction times at mid age. This points to a possible speed and/or accuracy tradeoff to maintain enhanced accuracy at mid age.

On the covert attention task, performance levels were no different between mid aged e4+ and e4−. This again contrasts with young adult performance on this task, in which we have reported a reduced validity effect for e4+, and increased parietal activity on both congruent and incongruent trials (Rusted et al., 2013). At mid age, neural activity did not differentiate e4+ from e4− during this task. This may be an early marker of changed capacity to accommodate task demands; we develop this idea later in the discussion.

In the PM task, e4+ were significantly slower on both the ongoing task and PM trials, while showing a trend toward greater accuracy. Neural differences were observed on PM trials, with mid aged e4+ showing less activity in extrastriate and parietal regions, relative to e4−.

The antagonistic pleiotropy hypothesis would suggest a transition point for e4+ as they move from being cognitively advantaged in young adulthood to cognitively disadvantaged in older adulthood (Han and Bondi, 2008). The evidence presented here suggests that behavioral advantages in e4+ might persist at age 45–55 years, although such advantages are reduced relative to young adulthood, at least in the specific cognitive domains tested here. Indeed, results suggested a speed and/or accuracy tradeoff operating in e4+, suggesting that e4+ might be investing more effort, or using different strategies, to maintain performance. This could reflect a trajectory of earlier cognitive decline in e4+ that negates early life advantages by mid age.

A number of imaging studies have explored APOE-related neural differences across the life span. Young adult e4+ show enhanced resting state activations and greater hippocampal activity during encoding memory tasks (Filippini et al., 2009), as well as higher scores on measures of axial diffusivity, an index of white matter integrity, which correlated with cognitive performance measures (Rusted et al., 2013). Conversely, diminished cerebral metabolic rates for glucose have been demonstrated in young and mid-aged e4+ (Reiman et al., 2004). Relative to younger individuals, mid-aged e4+ show decreases in temporal and cerebellar activity, suggesting that e4+ fail to recruit normal age-related compensatory processes (Filippini et al., 2011). In older adults, the e4 allele has been associated with decreased volume and disrupted white matter integrity in hippocampal regions (Honea et al., 2009), and a greater BOLD response in multiple brain regions during learning tasks (Bondi et al., 2005; Han et al., 2007). These results suggest that the e4 allele influences age-related neural changes. The evidence suggests e4+ may possess structural and metabolic differences from early in life, which then affect how compensatory strategies are implemented in response to aging.

To investigate neural changes from youth to mid-age, we contrasted fMRI data from our mid age cohort with that acquired in a group of young adults under an identical protocol (Evans et al., 2013b; Rusted et al., 2013). In both covert attention and PM tasks, mid-age participants showed greater activity across a number of brain regions. Neural differences at mid age were investigated, and a masking strategy used to isolate genotype-specific differences in activation between the young and mid-aged groups.

On PM trials, mid aged e4+ showed decreased extrastriate activity relative to mid-aged e4−. An impaired extrastriate response in mid aged e4+ has been reported elsewhere (Evans et al., 2013a), and could index accelerated neural aging: elderly adults reliably show decreased occipital lobe activity (across a variety of cognitive paradigms), assumed to reflect impaired sensory processing (Cabeza et al., 2004, Madden, 2007). We also found that extrastriate activity showed stronger modulation by reaction time in e4− than in e4+. Thus, at mid age, it would appear that early visual regions are more actively engaged in processing a PM response in e4−, while e4+ must invoke other strategies to process the PM cue. Relative to young adults, mid-age e4+ showed additional recruitment of left inferior frontal regions (extending into left orbitofrontal cortex) and left parahippocampus. These changes were not present in e4−. Left inferior frontal and oribitofrontal regions have been implicated in PM (Rusted et al., 2011; Simons et al., 2006), as has left parahippocampus (Kalpouzos et al., 2010). Frontal contributions to PM likely reflect the operation of executive control systems, which integrate external cues with stored *internal representations* to guide behavior (Burgess et al., 2011). These results suggest that e4+ show greater recruitment of PM-relevant frontal regions as they move into mid age, possibly to compensate for early visual processing difficulties. Offering direct support for this suggestion, a correlation between activity in left inferior frontal and PM accuracy was present in e4+, but not e4−. Although a Fisher transform showed no difference in the r-values of the correlations between the 2 groups, this should not detract from the substantive correlation demonstrated between performance and activity in e4+; this correlation did not approach significance in the e4− group data. Greater recruitment of parahippocampus could also form part of a compensatory response in e4+, but because no correlations were found between activity in this region and cognitive performance, this finding is harder to interpret. Hippocampal regions are not normally engaged by the PM task used here (Rusted et al., 2011), but some studies have shown hippocampal overactivation in e4+ that is not task-specific (Trachtenberg et al., 2012), which concurs with this finding.

In contrast, the e4 allele was associated with decreased activity in parietal regions. Mid-aged e4+ showed diminished recruitment of left SPL compared with youth, and this was not present in e4−. Mid-aged e4+ also showed decreased left SPL activity relative to mid-aged e4−. Parietal activity is commonly reported during PM tasks (Burgess et al., 2011; Simons et al., 2006), and SPL forms part of the dorsal attentional network, associated with externally directed cognition (Corbetta and Shulman, 2002). Connectivity between left SPL and lateral frontal and orbitofrontal regions has been reported (Vincent et al., 2008), suggesting that these regions might work in tandem to direct attention to the PM cue according to task instructions. The results here suggest that e4+ show diminished SPL, but enhanced frontal activity, as they move from youth to mid age. Enhanced frontal activity could reflect a compensatory strategy, and the performance effects (slower responding but a trend to higher accuracy) point to a more executive, top-down, response style in mid aged e4+. This was confirmed by the finding that left inferior frontal activity correlated with accuracy in e4+ only. Diminished parietal response could impair detection of the PM cue, but enhanced frontal activity could reflect a stronger representation of the PM intention, compensating for less effective low-level processing of the cue.

The covert attention task showed similar findings. Mid age cognitive and neuroimaging data revealed no effects of genotype, but genotype differences emerged when comparisons were made with imaging data from the young adult group. Mid-age e4− showed age-related increases in activity in inferior and superior parietal areas, whereas e4+ showed no such increases. This mirrors previous work showing a failure of normal age-related compensatory activity in e4+ (Filippini et al., 2011). Because young adult e4+ show greater parietal activity than their e4− peers during this task (see Rusted et al. (2013) and parameter estimates in Fig 3C), it could be that e4+ simply have no more compensatory capacity to exploit as they move into mid age. Given that all volunteers showed actual declines in parietal activity on the PM task by mid age, however, it is more likely that e4+ are experiencing early pathologic changes in parietal structures that are interfering with recruitment of those structures. Healthy older e4+ have a greater burden of amyloid pathology (Caselli et al., 2010), and increased brain amyloid beta levels are associated with faster declines in parietal metabolism (Ewers et al., 2012). Furthermore, the increased amyloid burden and metabolic dysfunction observed in early-onset AD have a strong parietal focus (Ossenkoppele et al., 2012). Thus, parietal structures might be particularly vulnerable to amyloid deposition in e4+ as they age, and this could be exacerbated by functional overactivations in these regions in youth (Jagust and Mormino, 2011).

Enhanced frontal activity in e4+, observed in the PM, but not the covert attention task, could reflect early implementation of a compensatory strategy used by e4+ in response to parietal pathology. Healthy elderly e4+ have diminished gray matter regional volumes in hippocampal and parietal regions, but a trend toward increased regional volumes in frontal cortex (Honea et al., 2009). This would be consistent with a premature aging model, because greater recruitment of frontal areas has been associated with the maintenance of cognitive performance in older adulthood (Cabeza et al., 2002).

## 5 Conclusions

We have shown that on tests of attention and PM, mid-age e4+ perform as well or better than their e4− peers. On the RVIP and PM tasks, there was some evidence of a speed and/or accuracy tradeoff in e4+, suggesting that by this age, the e4+ group are working harder or changing their strategy, to sustain the same level of performance as e4−. Comparing fMRI data with that acquired from a young adult sample, we found diminished age-related parietal recruitment, and enhanced age-related frontal activity in e4+. This is in line with the speed and/or accuracy tradeoff demonstrated by the cognitive performance data, and suggests a more executive, top-down processing strategy in mid-aged e4+. These neural changes suggest a compensatory mechanism operating in mid-aged e4+ to support impaired parietal activation in response to task demands. Future studies are needed to explore whether these cognitive and functional activation changes are linked to genotype-specific neuronal changes, and particularly amyloid deposition, in this region. This work is the first to demonstrate that mid-aged e4+ show patterns of neural activity reminiscent of those usually seen in much older adults, consistent with the notion of accelerated neural aging in this group.

## Disclosure statement

Naji Tabet has received research grants (unrelated to this study) from Pfizer and Novartis. He also received consultation and/or speaker fees from Pfizer, Eli Lilly, AstraZeneca, Shire, Novartis, and Lundbeck. The other authors have no conflicts of interest to disclose.

## Figures and Tables

**Table 1:** Volunteer characteristics and measures of baseline cognitive performance (standard deviations in parentheses)

Group	Age, years	Gender	IQ	Episodic memory (recalled words, max 20)	Education, years
Young adult group					
e4+ (n = 21)	21.4 (2.2)	13 F	113 (4)	9.0 (2.7)	15.1 (0.2)
e4− (n = 20)	20.9 (1.4)	14 F	115 (3)	9.7 (2.9)	15.1 (0.3)
t statistic	t = 0.82		t = 0.02,	t = 0.42	t = 0.18
	*p* = 0.42		*p* = 0.23	*p* = 0.42	*p* = 0.56
Mid-age group					
e4+ (n = 19)	49.4 (3.9)	12 F	119 (5)	6.9 (2.3)	15.0 (1.55)
e4− (n = 21)	50.5 (4.5)	11 F	121 (4)	7.9 (2.1)	14.6 (1.57)
t statistic	t = 0.85		t = −1.97	t = 1.44	t = 0.75
*p* value	*p* = 0.39		*p* = 0.10	*p* = 0.16	*p* = 0.51

**Table 2:** Performance on RVIP and covert attention tasks by genotype. Standard deviations shown in brackets

Age	Genotype	RVIP task			Covert attention task		
		Mean detections per min (maximum 8)	Mean false alarms per min	RT (ms) to correct detections	RT (ms) to validly cued trials	RT (ms) to invalidly cued trials	Validity effect (ms)
Young	e4+	5.46 (1.42)	0.46 (0.29)	494 (46)	358 (50)	387 (58)	26 (5.5)
	e4−	4.57 (1.15)	0.88 (0.84)	526 (102)	329 (72)	371 (67)	42 (5.4)
Mid	e4+	5.48 (1.55)	0.52 (0.37)	537 (77)	422 (84)	476 (108)	54 (44)
	e4−	5.25 (1.47)	0.56 (0.58)	512 (63)	393 (90)	457 (78)	64 (25)

Key: RVIP, rapid visual information processing; RT, reaction time.

**Table 3:** Performance on PM task tasks by genotype. Standard deviations shown in brackets

Age	Genotype	PM task			
		PM detections (maximum 32)	PM RT (ms)	Ongoing detections (maximum 192)	Ongoing RT (ms)
Young	e4+	26.1 (4.3)	708 (73)	180.7 (8.8)	625 (84)
	e4−	26.7 (3.6)	674 (82)	180.6 (7.6)	613 (87)
Mid	e4+	27.6 (3.6)	802 (104)	184 (5.67)	737 (94.4)
	e4−	25.1 (4.90)	702 (140)	180 (8.46)	653 (112.4)

Key: PM, prospective memory; RT, reaction time.
