# Supplementary material for: Cognitive and neural signatures of the APOE E4 allele in mid-aged adults
Source: Neurobiol Aging. 2014 Jul;35(7):1615–23. doi: 10.1016/j.neurobiolaging.2014.01.145 (PMC4001126; doi:10.1016/j.neurobiolaging.2014.01.145)
Supplement: Supplementary Data [file mmc1.doc]

**Supplementary materials**

**fMRI recording and analysis**

To minimise signal artefacts originating from the sinuses, axial slices were tilted 30° from inter-commissural plane. Thirty-six 3 mm slices (0.75 mm interslice gap) were acquired with an in-plane resolution of 3 mm × 3 mm (TR = 3300 ms per volume, TE = 50 ms). Images were pre-processed using SPM8. (http://www.fil.ion.ucl.ac.uk/spm/). Raw T2-weighted volumes were spatially realigned and unwarped, spatially normalised to standard space and smoothed (8 mm kernel). **PM task:** Card order was pseudo-randomised, and permuted to maximise estimability of each event type (Frist*on et* al, 1999) while ensuring a delayed onset and minimum separation of PM events. Specifically, the constraints were that PM cards never occurred in the first 7 cards of the entire sequence, and that there were always at least 3 intervening cards between PM events. Since each deck remained independent of the next, we also required that one of the 4 PM trials per deck occurred within each quarter (13 cards). For each subject, three trial types (sort, withhold and PM) were modelled in first level design matrices (each session was analysed in a separate design matrix) that also included 6 movement parameters estimated during pre-processing. For Sort and PM trials, we only included trials where correct responses were made (first button response only). In a separate analysis, the reaction time for each Sort and PM trial was included as a parametric modulator, and modeled as a separate column in the design matrix. Genotype and condition (sort, withhold and PM and, in the reaction time analysis, sort/PM modulated by reaction time) were then entered into a second level full factorial model for the estimation of group effects. Gender was entered as a covariate. For the investigation of age-related effects, genotype (e4+,e4-), condition (sort, withhold, PM) and age (young, mid) were entered into a second level full factorial model. **CA task:** For each subject, three trial types (congruent, incongruent, and catch) were modelled in first level design matrices that also included 6 movement parameters estimated during pre-processing. For congruent and incongruent trials, we only included trials where correct responses were made (first button response only), and events were time-locked to presentation of the target. Genotype (e4+,e4-) and condition (congruent, incongruent) were then entered into a second level full factorial model for the estimation of group effects. To investigate age-related effects, a first level contrast was used to calculate activity associated with the validity effect, contrasting congruent and incongruent trials. Genotype (e4+,e4-) and age (young, mid) were then entered into a second level full factorial model.
